# Leveraging agricultural waste manipulation using a novel resveratrol-producing endophytic fungus, *Cladosporium sphaerospermum* AH26: optimization and enzymatic investigation

**DOI:** 10.3389/fbioe.2026.1933402

**Published:** 2026-09-16

**Authors:** Amal A. Al Mousa, Adel Eltoukhy, M. A. Abo-Kadoum, Omar M. Atta, Khalifa S. H. Eldiehy, Hassan Mohamed, Ahmed Abdel-Hadi, Nageh F. Abo-Dahab, Abdel-Rehim A. El-Shanawany, Abdallah M. A. Hassane

**Affiliations:** 1 Department of Botany and Microbiology, College of Science, King Saud University, Riyadh, Saudi Arabia; 2 Department of Botany and Microbiology, Faculty of Science, Al-Azhar University, Assiut, Egypt; 3 Colin Ratledge Center for Microbial Lipids, School of Agricultural Engineering and Food Science, Shandong University of Technology, Zibo, China

**Keywords:** agricultural wastes, *Cladosporium sphaerospermum*, fermentation, optimization, response surface methodology, resveratrol

## Abstract

Resveratrol (RES) is utilized in multiple pharmaceutical and industrial applications. Rising global demand has required immediate novel production solutions for sustainable RES supply. Endophytic fungi are a promising source for RES production; however, their conventional culture impedes economic viability. This study aimed to leverage agricultural wastes as a low-cost carbon source for improving RES productivity alongside the associated hydrolytic enzymatic activities using grapevine-derived endophytic fungus *Cladosporium sphaerospermum* AH26. *C. sphaerospermum* was isolated from the stem tissues of grapevine (*Vitis vinifera* L. Starlight cultivar) and identified by internal transcribed spacer sequencing. The RES-producing capability of AH26 was assessed on potato dextrose broth medium (PDB) and on different agricultural wastes, such as banana peel, wheat straw, and sugarcane bagasse. Thin-layer chromatography (TLC) and high-performance liquid chromatography (HPLC) were employed for the qualitative and quantitative analysis of RES production, respectively. Hydrolytic enzyme activities were initially screened on agar plates and subsequently quantified using a dinitrosalicylic acid-based assay. Optimization of cultivation conditions for maximizing RES productivity on agricultural wastes was achieved through response surface methodology (RSM) using Box–Behnken design. *C. sphaerospermum* showed significant efficacy for RES production when cultivated on PDB, banana peel, wheat straw, and sugarcane bagasse, yielding total concentrations of 42.30 µg/mL, 41.78 µg/mL, 39.59 µg/mL, and 40.33 μg/mL, respectively. RSM optimization of agricultural waste cultivation conditions resulted in 2.7-, 3.4-, and 4.7-fold increases in RES concentration on banana peel, wheat straw, and sugarcane bagasse, respectively. Analysis of variance revealed that the interaction between temperature and other cultivation parameters, including incubation period, pH, and substrate concentration, significantly affected RES production. The analysis of hydrolytic enzymes demonstrated a positive correlation between enzymatic activity and RES productivity by *C. sphaerospermum*. Our findings identified *C. sphaerospermum* AH26 strain as a promising novel and efficient strain for RES production and demonstrated the potential of agricultural waste-based cultivation as a viable economic and sustainable strategy for the cost-effective production of RES by mycoendophytes.

## 1 Introduction

Resveratrol (3,5,4′-trihydroxystilbene, Figure 1) is a well-known secondary metabolite that is synthesized in some plants as a naturally occurring phytoalexin *via* the phenylpropanoid pathway (Lu et al., 2016; Abo-Kadoum et al., 2022; Bejenaru et al., 2024; Hemdan and El-Shiekh, 2025). Resveratrol (RES) belongs to the stilbenes group, consisting of two benzene rings connected by an ethylene bridge (Bejenaru et al., 2024). It was first detected in the roots of the white hellebore plant (*Veratrum grandiflorum*) in 1939 (Takaoka, 1940). RES is synthesized endogenously in significant concentrations by diverse plants as a defense mechanism against various biotic attacks, such as fungal and bacterial pathogens, and abiotic stressors, such as UV radiation and mechanical wounding (Dwibedi and Saxena, 2022). This polyphenolic compound is abundant in various plant types like grapes (*Vitis vinifera*), berries, peanuts (*Arachis hypogaea*), chocolate, tea, and cassia seeds (Burns et al., 2002; Baur and Sinclair, 2006; Wang et al., 2013; Mei et al., 2015; Tian and Liu, 2020; Govindaraj, 2026). Among these botanical sources, grapes are reported as the primary source of RES (Al-Jubouri et al., 2021; Căpruciu and Gheorghiu, 2025; Karagül and Uğraş, 2025).

**FIGURE 1:**
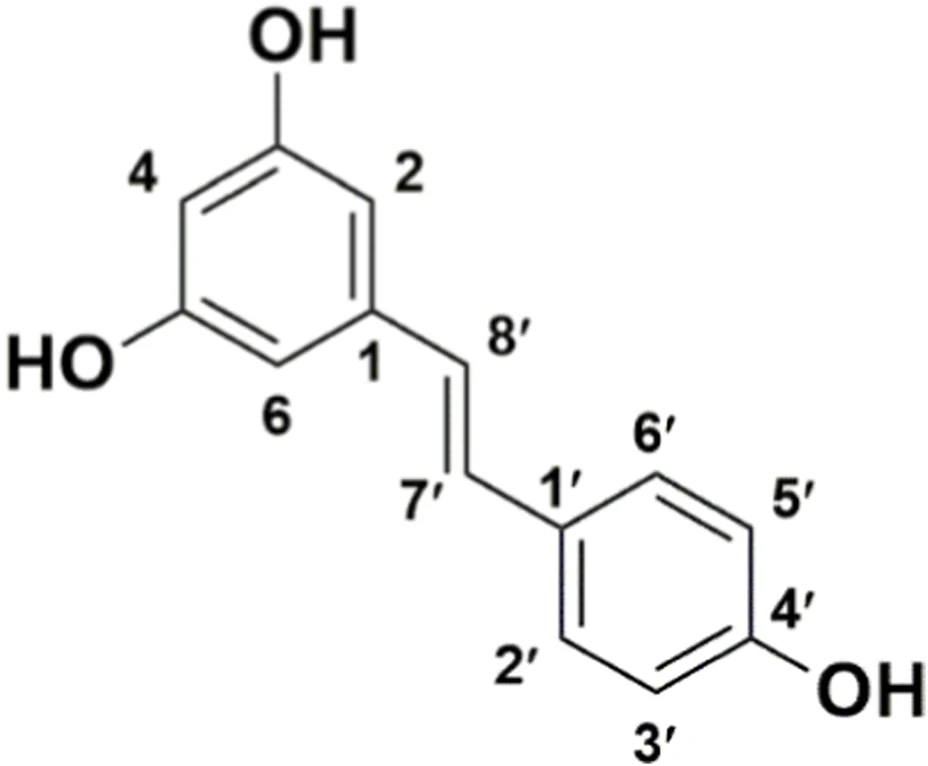
Chemical structure of resveratrol.

The detection and quantification of resveratrol are essential for evaluating its biosynthesis, extraction efficiency, and biological activities. Several analytical techniques have been employed for RES analysis, including high-performance liquid chromatography (HPLC), liquid chromatography–mass spectrometry (LC–MS/MS), gas chromatography–mass spectrometry (GC–MS), and capillary electrophoresis (CE) (Căpruciu and Gheorghiu, 2025; Goel et al., 2025).

Due to its therapeutic properties, RES was extensively used in traditional Chinese medicine as a botanical source for therapy (Prakash et al., 2024). Recently, RES has been evaluated as beneficial for treating numerous diseases such as foodborne infections, metabolic disorders (diabetes and obesity) (Capozzi et al., 2022), cardiovascular diseases (CVDs) (Godos et al., 2024), Alzheimer disease (Gui et al., 2024), neurological disorders (Robb and Stuart, 2010), liver dysfunction, and many types of cancer (Kaur et al., 2022; Filipe et al., 2023). Due to the significant role and therapeutic applications of RES, considerable attention has been paid to finding alternative sources, in addition to maximizing its production rate (Costa et al., 2025). Conventionally, RES was produced from plants; however, this process was limited owing to seasonal cultivation and low productivity (Kiselev, 2011). The implementation of microbial cell factories offers a promising tool for enhancing RES biosynthesis under tightly regulated metabolomics and fermentation parameters (Xu et al., 2024; Zhang et al., 2025).

Fungi, particularly endophytes, are the most common microorganisms intensively used as an alternative source for RES biosynthesis to meet the global demand (Dwibedi and Saxena, 2019b; Lu et al., 2021; Meshram et al., 2022). As internal partners with plant tissue, endophytic fungi are a significant reservoir of novel bioactive metabolites in addition to enhancing the growth and stress tolerance of host organisms through co-evolution and horizontal gene transfer (Abo-Kadoum et al., 2022). The utilization of available cost-effective and sustainable substrates is essential to produce valuable metabolites through microbial fermentation. In this context, agricultural wastes are promising substrates for microbial fermentation owing to their abundance, low cost, and sustainability (Oluwaseun et al., 2025; Wani et al., 2026). These untapped resources contain the necessary energy and moisture required for microbial growth and reproduction (Oluwaseun et al., 2025). Among these agricultural wastes, banana peel is a common waste product, as banana is among the most consumed fruits in the world (Naik et al., 2023). Industrial production of sugars and their derivatives generates large amounts of byproducts known as sugarcane bagasse, which has a high content of sugars and vitamins required for microbial growth in biotechnological processes to produce high-value products (Wanderley et al., 2013; Kamboj et al., 2024). Wheat straw stands as a ubiquitous agricultural residue, rich in carbohydrates including cellulose, xylan, and starch (Zhu et al., 2024). These agricultural wastes have been extensively studied by many researchers in numerous fermentation processes to generate cost-effective and valuable products (Zhu et al., 2024).

The utilization and bioconversion of agricultural wastes by microorganisms depend on the production of enzymes that degrade the complex polysaccharides into simple sugars to facilitate fungal growth and biosynthesis of valuable metabolites (Dhiman et al., 2024; Pérez-Contreras et al., 2025). Among these enzymes, cellulases, xylanases, pectinases, and amylases play an important role in supporting endophytic fungi in agricultural waste bioconversion and production of valuable bioactive compounds, including RES (Huang et al., 2024).

The microorganism fermentation process is highly influenced by operational conditions such as temperature, pH, inoculum size, incubation period, and substrate concentration, and it must be optimized to maximize the production rate. Traditionally, the one-factor-at-a-time (OFAT) optimization approach, which involves changing one factor while others are fixed, is extensively used (Taylor et al., 2023). This kind of optimization has numerous disadvantages, such as being time consuming and ignoring the interaction influence between variables (Taylor et al., 2023; Moon et al., 2024). These disadvantages can be solved by using statistical optimization techniques. Unlike OFAT, response surface methodology (RSM) is extensively used by researchers as an alternative to the OFAT approach, which evaluates the interaction between different variable levels and reduces time consumption (Khorsheed et al., 2025). In this study, the grape-derived fungus *C. sphaerospermum* AH26 was isolated and identified as a potent RES producer on different agricultural wastes. RES productivity was further enhanced through the RSM approach.

## 2 Materials and methods

### 2.1 Plant material and fungal isolation

The endophytic fungus *C. sphaerospermum* was isolated from the stem tissues of the grapevine *V. vinifera* cultivar Starlight and identified. The fungal endophytes were isolated following the standard protocol developed by Dwibedi and Saxena (2019a). The plant materials were washed with sterile distilled water to remove any debris and dust on the surface. Then, they were cut into small segments with a sterile scalpel and immersed in 70% ethanol for 30 s, then treated with 1% sodium hypochlorite for 2 min. After that, the segments were washed three times with sterile distilled water and inoculated on potato dextrose agar (PDA) plates under aseptic conditions. The Petri plates were incubated at 28 °C ± 2 °C for 7 days. The growing fungal hyphae were transferred to fresh PDA plates for purity of the cultures and subcultured repeatedly under the same conditions. The purified isolates were preserved in 20% glycerol at −20 °C for further studies.

### 2.2 Molecular identification of the fungus

The fungal isolate was purified and cultivated on PDA plates at 28 °C for 3–4 days. Fresh mycelial biomass was harvested and ground into fine powder in liquid nitrogen (Abdelrahem et al., 2024). The powdered mycelium was used to extract genomic DNA using the DNAquick Plant System Isolation Kit (Tiangen Biotech Co., Ltd., Beijing, China) according to the manufacturer’s instructions. The purified DNA was eluted in the supplied buffer and stored at −20 °C until further analysis (Shah et al., 2021). Molecular identification of the fungal isolate was performed by amplification of the ribosomal DNA (rDNA) region using the universal fungal primers ITS1 and ITS4. The ITS regions of the rRNA gene were amplified by polymerase chain reaction (PCR) using universal fungal primers ITS1 (5′-TCC​GTA​GGT​GAA​CCT​GCG​G-3′) and ITS4 (5′-TCC​TCC​GCT​TAT​TGA​TAT​GC-3′) (Sangon Biotech, Shanghai, China). PCR amplification was carried out using 2× Accurate Taq Master Mix (QIAGEN, Germany). Each reaction was performed in a total volume of 25 μL containing approximately 50 ng of template DNA, 12.5 μL of 2× PCR Master Mix, 0.5 μL of each primer, and nuclease-free water to the final volume (Mohamed et al., 2021).

Amplification was performed in a thermal cycler (Mastercycler®, Eppendorf, Germany) under the following conditions: initial denaturation step at 95 °C for 10 min; 35 cycles of denaturation at 95 °C for 30 s, annealing at 56 °C for 30 s, extension at 72 °C for 1 min 45 s, and final extension at 72 °C for 3 min 30 s. PCR products were analyzed by electrophoresis in 1% (w/v) agarose gels in Tris-borate-EDTA buffer and visualized with a gel documentation system (Syngene, United States). The amplified fragments were purified with a QIAquick Gel Extraction Kit (Omega Bio-Tek) according to the manufacturer’s instructions. Purified amplicons were sequenced by Sangon Biotech (Shanghai) Co., Ltd. on a commercial basis. The obtained sequences were compared to reference sequences from the National Center for Biotechnology Information (NCBI) nucleotide database using the Basic Local Alignment Search Tool (BLAST) algorithm to determine the closest phylogenetic affiliation of the isolate (Mohamed et al., 2021).

#### 2.2.1 Phylogenetic analysis

The obtained ITS sequences were submitted to NCBI’s GenBank database, and respective accession numbers were assigned. Closely related reference sequences were identified by a BLAST sequence similarity search against the NCBI nucleotide database (http://www.ncbi.nlm.nih.gov/BLAST/). Homologous sequences were selected from GenBank for phylogenetic analysis. Multiple sequence alignment was conducted using Clustal Omega as implemented in MEGA X (Version 11.0.13) (Tamura et al., 2021). Phylogenetic construction was performed based on the aligned ITS sequences using the neighbor-joining (NJ) method. The evolutionary distances were computed using the p-distance model. The robustness of the inferred tree topology was tested by bootstrap analysis with 1000 replicates (Saitou and Nei, 1987).

### 2.3 Detection of RES

The endophytic fungus *C. sphaerospermum* was aseptically inoculated with a 5 mm mycelial plug into autoclaved 100 mL of PDB medium, which was dispensed into 250 mL Erlenmeyer flasks and inoculated aseptically with one 5-mm actively growing mycelial plug taken from the margin of a 7-day-old culture grown on PDA. To standardize the inoculum, plugs were obtained from cultures of the same age and physiological status using a sterile cork borer and incubated in a rotary shaker at 150 rpm and 28 °C for 7 days. The culture filtrate was prepared following incubation with Whatman filter paper No. 1. The culture supernatant was extracted with an equal volume of ethyl acetate (EtOAc) twice. The organic layer was dried and redissolved in methanol (Dwibedi et al., 2019). RES was qualitatively screened in the crude EtOAc extract by TLC and confirmed using HPLC analysis. The TLC plate (20 × 20 cm) was spotted with 10 µL of the crude extract of the endophytic fungus *C. sphaerospermum* and a standard RES solution (5 μg/mL). All spots were developed on the plate with a mobile phase of EtOAc:methanol:water in the ratio of 8:1:1, v/v, respectively. The separated bands were visualized under UV light, and the characteristic blue fluorescence spot of RES was compared with the crude extract of *C. sphaerospermum*. The rate of flow (Rf) values of the two comparable spots were determined and compared. Rf is calculated by dividing the distance from the center of the blue spot to the baseline by the distance moved by the mobile phase to the baseline (Liu et al., 2016).

The quantitative measurement of RES in the EtOAc crude extract was performed using an Agilent 1260 Infinity II HPLC system equipped with a quaternary pump, an autosampler, a temperature-controlled column compartment, and a diode array detector (DAD). Separation was achieved on an EC-C18 column (150 × 4.6 mm, 5 µm particle size, Agilent Technologies) maintained at 30 °C. A C18 guard column (10 × 4.0 mm, 5 µm particle size, Agilent Technologies) was installed prior to the analytical column. The injection solvent was the mobile phase at the initial gradient composition. The DAD was set to detect at a wavelength of 320 nm. The mobile phase consisted of acetonitrile in water (50:50) (eluent A) and 0.1% formic acid in water (eluent B). The flow rate was maintained at 0.4 mL/min, and the injection volume was 5 µL. The linear gradient elution program was optimized as follows.

**Table:** 

Time (min)	% eluent A	% eluent B	Change rates for the mobile phase composition	Transition type
0.0	90	10	0%	Initial equilibration
10.0	90	10	3.5% per min	Isocratic hold
35.0	5	95	8.5% per min	Linear gradient
45.0	90	10	0%	Linear gradient
55.0	90	10	0%	Post-run re-equilibration

The limit of detection (LOD) was 0.01 μg/mL, and the limit of quantification (LOQ) was 0.05 μg/mL, with a linearity regression equation y = 5.36242e6 x + 16,239.23050 (*R* ^2^ = 0.9967) (Al Mousa et al., 2026).

### 2.4 Preliminary enzyme screening

#### 2.4.1 Screening on agar plates

The production of hydrolytic enzymes was tested on Czapek’s agar medium containing (g/L): KH_2_PO_4_, 1.0; NaNO_3_, 2.0; MgSO_4_·7H_2_O, 0.5; and CaCl_2_·2H_2_O, 0.5. Starch, carboxymethyl cellulose, pectin, and xylan were supplemented as carbon sources for the synthesis of amylase, cellulase, pectinase, and xylanase, respectively, at 10 g/L. The pH was adjusted to 7.0, and the inoculated plates were incubated at 28 °C ± 2 °C for 5 days. After that, the enzyme production was determined according to the method of Meddeb-Mouelhi et al. (2014). After incubation, the agar plates were flooded with iodine solution for 15 min and observed for the development of clear zones around the fungal colonies, indicating the enzymatic degradation of the respective substrates.

#### 2.4.2 Screening by submerged fermentation

The untreated substrates were washed with distilled water to remove adhering impurities, dried at 65 °C for 24 h, and milled to a fine powder. No chemical, thermal, or biological pretreatment was performed before their use. The resulting powdered substrates were used at a concentration of 10 g/L in Czapek’s mineral salts broth. The substrates were banana peel, sugarcane bagasse, and wheat straw brought from Assiut City, Egypt. The pH of the medium was adjusted to 7.0 before sterilization by autoclaving. The strains were inoculated in Erlenmeyer flasks (250 mL) with 100 mL of the broth medium and with spore suspensions (10^7^ spores/mL). Spore concentration was determined using a Neubauer hemocytometer by counting spores and subsequently diluting the suspension with sterile distilled water to obtain the desired concentration. The inoculated flasks were incubated for 7 days at 28 °C ± 2 °C with continuous shaking at 120 rpm. Following incubation, the cultures were centrifuged, and supernatants were collected and stored for subsequent enzymatic analysis (Al Mousa et al., 2022a; El-Sayed et al., 2024).

#### 2.4.3 Quantitative screening of RES with amylase, cellulase, pectinase, and xylanase on agricultural wastes

Amylase, cellulase, pectinase, and xylanase activities were measured using the dinitrosalicylic acid (DNS) method according to Miller (1959). Amylase activity was determined by adding enzyme supernatant to 0.5 mL `1, carboxymethylcellulose, pectin, and xylan (1% w/v) in an acetate buffer (50 mM, pH 5.0). The mixture was kept at 50 °C for 30 min, then 1 mL of 3,5-DNS acid reagent was added to terminate the reaction, and the mixture was incubated at 100 °C for 10 min. After cooling, the absorbance was measured at 570 nm using a spectrophotometer (Jenway 7315, United Kingdom). The amount of reducing sugars was estimated by plotting the calibration curve using glucose as a standard. The results were presented as the mean value, and all tests were run in triplicate. One unit was defined as the amount of the enzyme in 1 mL required to release 1 μmol of reducing sugar from a substrate within 60 s under the most favorable experimental conditions. The quantity of reducing sugar released during the 30 min incubation was normalized to the reaction time when calculating enzyme activity (Fatokun et al., 2016). Using HPLC analysis, the quantity of RES produced on each substrate was quantified.

### 2.5 Optimization of RES productivity on different substrates under submerged fermentation

The Box–Behnken design (BBD) of RSM was used to optimize the factors that led to the increase in RES yield. These factors were A, temperature; B, pH; C, inoculum size; D, incubation period; and E, substrate concentration (Table 1). The pH was adjusted to the desired values with a buffer system consisting of glycine-NaOH buffer (0.05 M, pH 9.0), potassium phosphate buffer (0.05 M, pH 7.0), and sodium acetate buffer (0.05 M, pH 5.0). Forty-six experiments with the central points satisfied the polynomial pattern based on a BBD (five variables) generated by Minitab 18 software (Version18.1.1.0) (Al Mousa et al., 2022a; Al Mousa et al., 2022b). The number of tests (N) for a three-level (−1, 0, and +1) and five-factor experimental BBD was determined using the subsidiary equation as follows:

$$N=2k\left(k-1\right)+C_{0},$$ (1)

where k is the number of factors and C_0_ is the number of central points, which is equal to 6.

**TABLE 1:** Box–Behnken design levels of independent factors.

No.	Factor symbol	Variable	Units	Range		
				Minimum (−1)	Maximum (+1)	Mean (0)
1	A	Temperature	°C	20	40	30
2	B	pH	-	5	9	7
3	C	Inoculum size	mL	1	5	3
4	D	Incubation period	day	5	15	10
5	E	Substrate concentration	g/100 mL	1	10	5.5

The influence of variables on the simulation (Y) was interpreted through a second-order polynomial equation, designed to predict the optimum conditions for RES production. The amounts of detected RES were estimated by plotting the calibration curve using standard RES.

$$Y=\beta_{0}+\beta_{A}A+\beta_{B}B+\beta_{C}C+\beta_{D}D+\beta_{E}E+\beta_{\text{AA}}A^{2}+\beta_{\text{BB}}B^{2}+\beta_{\text{CC}}C^{2}+\beta_{\text{DD}}D^{2}+\beta_{\text{EE}}E^{2}+\beta_{\text{AB}}\text{AB}+\beta_{\text{AC}}\text{AC}+\beta_{\text{AD}}\text{AD}+\beta_{\text{AE}}\text{AE}+\beta_{\text{BC}}\text{BC}+\beta_{\text{BD}}\text{BD}+\beta_{\text{BE}}\text{BE}+\beta_{\text{CD}}\text{CD}+\beta_{\text{CE}}\text{CE}+\beta_{\text{DE}}\text{DE},$$ (2)

where Y is the response variable; β_0_ is the intercept; β_A_, β_B_, β_C_, β_D_, and β_E_ are the linear coefficients; β_AA_, β_BB_, β_CC_, β_DD_, and β_EE_ are the square coefficients; β_AB_, β_AC,_ β_AD_, β_AE_, β_BC_, β_BD_, β_BE_, β_CD,_ β_CE_, and β_DE_ are the interaction coefficients, and A, B, C, D, E, A^2^, B^2^, C^2^, D^2^, E^2^, AB, AC, AD, AE, BC, BD, BE, CD, CE, and DE are the levels of independent variables. The respective coefficients of variables, interaction variables, and contour plots were obtained using Minitab 18 software. The regression equation was examined, and response plots were generated to determine the optimal values of the evaluated variables. The regression coefficient (*R* ^2^) was used to express the profit of the polynomial equation, and the F test was used to determine its statistical significance. Quantitative screening of RES using HPLC along with amylase, cellulase, pectinase, and xylanase produced on different agricultural wastes was carried out as described in Section 2.3 and Section 2.4.3.

### 2.6 Data analysis

Values were expressed as mean ± SD. Preliminary tests and measurements were performed in triplicate. Pearson’s correlation coefficients were calculated using SPSS software (Version 16). The experimental design and analysis of variance (ANOVA) for RSM-BBD were carried out using Minitab 18 software (Version 18.1.1.0).

## 3 Results

### 3.1 Molecular identification of *C. sphaerospermum*

The ITS sequence of the target strain AH26 produced a distinct band of approximately 500 bp, as revealed by agarose gel electrophoresis. The obtained molecular data showed strong agreement with the observed morphological characteristics. Sequence homology analysis of strain AH26, performed using the BLASTn tool against reference sequences available in the GenBank database, revealed that the isolate belongs to the genus *Cladosporium*, showing 100% sequence similarity with previously reported sequences of *C. sphaerospermum* (Figure 2). A phylogenetic tree based on the neighbor-joining method using the ITS sequence was constructed, illustrating the evolutionary relationship between the target strain and closely related taxa. The phylogenetic analysis further confirmed the placement of strain AH26 within the *C. sphaerospermum* clade. Therefore, the strain was identified as *C. sphaerospermum* AH26, and its sequence was deposited in the NCBI GenBank database with the accession number PX952784.

**FIGURE 2:**
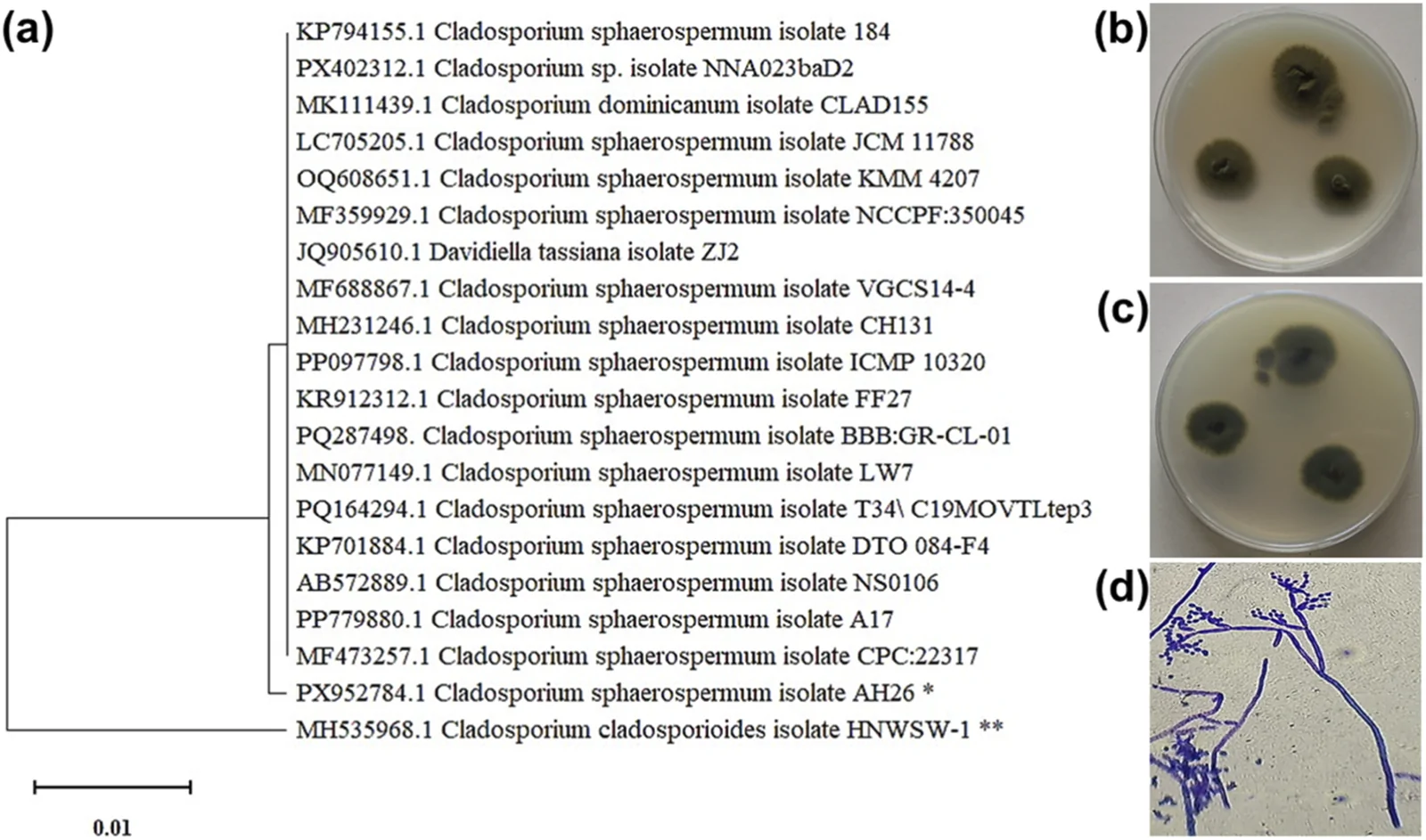
**(a)** Neighbor-joining (NJ) phylogenetic tree of *C. sphaerospermum* AH26 and related strains obtained from GenBank, constructed based on ITS sequences using MEGA X software (Version 11.0.13). ITS sequences were aligned using ClustalW, and the tree was constructed with 1,000 bootstrap replications. The tree scale (0.01) represents evolutionary distances in units of base substitutions per site calculated using the maximum composite likelihood method. AH26* (PX952784) was studied in this study, and HNWSW-1** was chosen as the outgroup strain. **(b)** Morphological features of the AH26 strain on PDA (obverse); **(c)** AH26 reverse; **(d)** AH26 studied by optical microscope X40.

### 3.2 Detection and quantification of RES

The endophytic fungus *C. sphaerospermum* was grown in PDB medium for 7 days. The fermentation broth was extracted with EtOAc, and RES quantification was performed by HPLC analysis. The strain AH26 produced 42.3 μg/mL of RES under the tested conditions.

### 3.3 Preliminary screening of RES and enzyme production on agricultural wastes

The preliminary qualitative screening for extracellular fungal amylase, cellulase, pectinase, and xylanase activities revealed that *C. sphaerospermum* AH26 was capable of producing these enzymes on solid assay media. Quantitative assay under SmF, using banana peel as a substrate, showed that the strain AH26 produced RES (41.78 μg/mL) along with extracellular amylase, cellulase, and pectinase activities of 5.41 U/mL, 4.86 U/mL, and 3.26 U/mL, respectively. In contrast, when wheat straw was used as the substrate, the strain AH26 produced RES (39.59 μg/mL) together with extracellular amylase, cellulase, and xylanase activities of 3.41 U/mL, 6.19 U/mL, and 4.61 U/mL, respectively, while pectinase activity was not detected. However, a RES yield of 40.33 μg/mL was obtained when sugarcane bagasse was used as a substrate, with no detectable enzymatic activities (Table 2).

**TABLE 2:** Resveratrol and enzyme production by *C. sphaerospermum* AH26 on different substrates.

Substrate	RES conc. (µg/mL)	Enzyme activity (U/mL)			
		Amylase	Cellulase	Pectinase	Xylanase
Banana peel	41.78 ± 0.81	5.41 ± 0.13	4.86 ± 0.28	3.26 ± 0.10	ND
Sugarcane bagasse	40.33 ± 1.01	ND	ND	ND	ND
Wheat straw	39.59 ± 0.55	3.41 ± 0.12	6.19 ± 0.04	ND	4.61 ± 0.15

ND: not detected.

The data are presented as the mean of three replicates (mean ± SD).

### 3.4 Response surface methodology for optimization of RES production

The independent factors with their respective levels used in the optimization of RES production are presented in Table 1. BBD of the independent factors, along with predicted and experimental values, are shown in Table 3. The prediction equation for RES production by *C. sphaerospermum* AH26 on banana peel was as follows:

$$Y\;\left(µg/\text{mL}\right)=-2-5.78A-14.5B-11.4C+14.16D+41.44E+0.225A^{2}-0.629B^{2}-1.033C^{2}+0.507D^{2}+0.306E^{2}+0.681\text{AB}+0.391\text{AC}-0.937\text{AD}-0.597\text{AE}-0.87\text{BC}+0.927\text{BD}-1.797\text{BE}+0.792\text{CD}+0.412\text{CE}-0.861\text{DE}.$$ (3)

**TABLE 3:** RES production by BBD of optimization variables (experimental and predicted) (µg/mL) on different substrates by *C. sphaerospermum* AH26.

Run order	Variables					RES concentration (µg/mL)					
						Banana peels		Sugarcane bagasse		Wheat straw	
	A	B	C	D	E	Exp	Pred	Exp	Pred	Exp	Pred
1	25	9	3	10	5.5	26.90	19.16	62.50	70.73	26.65	28.46
2	30	7	5	10	10	83.40	73.44	32.50	33.20	43.85	39.76
3	25	7	3	10	1	9.05	2.09	6.70	−11.62	45.45	35.57
4	30	7	5	15	5.5	57.00	54.49	48.35	56.26	49.85	53.70
5	30	5	3	10	10	110.75	103.11	33.80	45.95	23.85	34.69
6	30	9	3	15	5.5	44.30	48.84	61.50	55.13	57.90	63.41
7	30	7	3	10	5.5	44.50	39.88	38.20	43.78	31.20	37.78
8	25	7	3	5	5.5	24.15	28.19	73.65	76.17	49.65	49.17
9	30	7	5	5	5.5	27.55	35.36	67.85	72.96	16.40	23.41
10	30	5	5	10	5.5	40.15	45.34	60.40	55.91	14.85	6.42
11	25	7	5	10	5.5	20.40	29.06	12.10	14.41	12.05	13.17
12	25	7	3	10	10	91.00	91.49	50.60	44.13	26.20	31.72
13	30	9	5	10	5.5	13.95	14.16	54.85	53.37	3.25	8.25
14	30	7	1	10	10	67.65	72.99	39.00	45.80	64.00	59.39
15	30	7	3	15	10	67.85	72.29	22.75	22.40	117.90	109.02
16	30	7	3	10	5.5	35.35	39.88	50.20	43.78	41.00	37.78
17	30	5	3	15	5.5	56.10	54.51	92.60	77.55	61.90	61.52
18	30	7	1	5	5.5	50.85	58.15	91.75	81.86	32.30	30.04
19	35	9	3	10	5.5	44.85	42.57	118.30	126.51	42.20	46.69
20	30	9	1	10	5.5	32.60	28.09	48.79	50.09	1.70	16.16
21	35	7	5	10	5.5	41.00	46.69	169.60	165.42	50.25	50.57
22	30	7	3	15	1	40.16	48.49	22.60	48.22	29.95	44.13
23	30	7	1	15	5.5	48.64	45.62	60.30	53.20	75.00	69.58
24	25	5	3	10	5.5	57.30	56.99	21.75	23.85	14.50	10.62
25	35	7	3	10	10	59.80	74.44	121.55	128.64	97.00	118.16
26	30	7	1	10	1	17.65	17.85	7.05	7.27	11.30	6.00
27	35	7	3	10	1	31.55	38.73	131.45	126.68	18.52	24.28
28	35	7	3	5	5.5	97.40	84.81	176.95	172.43	73.00	55.00
29	30	9	3	10	1	9.00	16.35	21.55	8.34	9.30	−11.84
30	30	5	3	10	1	7.15	8.22	31.55	36.16	18.75	22.62
31	30	7	3	5	10	113.70	107.74	114.00	99.76	60.00	54.23
32	35	7	3	15	5.5	54.70	41.29	170.75	164.90	134.85	121.66
33	30	7	3	10	5.5	47.20	39.88	42.40	43.78	42.30	37.78
34	30	7	3	5	1	8.50	6.43	4.50	16.23	11.80	29.09
35	30	5	3	5	5.5	72.10	69.76	86.25	86.56	31.85	30.01
36	30	9	3	10	10	47.93	46.56	61.95	56.28	80.30	66.13
37	30	7	3	10	5.5	34.00	39.88	38.00	43.78	37.15	37.78
38	35	7	1	10	5.5	50.20	45.82	126.80	128.74	61.35	62.00
39	35	5	3	10	5.5	48.03	53.17	188.80	190.88	68.75	67.55
40	30	7	5	10	1	18.58	3.48	19.90	14.02	7.90	3.12
41	30	7	3	10	5.5	37.25	39.88	39.00	43.78	35.00	37.78
42	25	7	3	15	5.5	75.10	78.32	37.15	38.34	48.00	52.34
43	25	7	1	10	5.5	45.25	43.85	48.50	56.94	22.80	24.25
44	30	7	3	10	5.5	40.95	39.88	54.90	43.78	40.00	37.78
45	30	5	1	10	5.5	44.85	45.33	66.75	65.04	20.00	21.02
46	30	9	3	5	5.5	23.20	26.99	82.50	91.49	21.05	25.10

Exp., experimental; Pred., predicted.

The output of RES by AH26 on sugarcane bagasse was fitted using the following equation:

$$Y\;\left(µg/\text{mL}\right)=1168-88.5A+38.1B-66.1C-18.33D+37.84E+1.890A^{2}+2.991B^{2}+0.088C^{2}+0.877D^{2}-0.942E^{2}-2.781\text{AB}+1.980\text{AC}+0.303\text{AD}-0.598\text{AE}+0.78\text{BC}-0.684\text{BD}+1.060\text{BE}+0.299\text{CD}-0.538\text{CE}-1.215\text{DE}.$$ (4)

The RES yield by AH26 on wheat straw was fitted using the following equation:

$$Y\;\left(µg/\text{mL}\right)=496-31.9A+57.6B+18.6C-33.86D-44.56E+0.502A^{2}-2.998B^{2}-3.205C^{2}+0.769D^{2}+0.104E^{2}-0.967\text{AB}-0.009\text{AC}+0.635\text{AD}+1.086\text{AE}+0.42\text{BC}+0.170\text{BD}+1.831\text{BE}-0.231\text{CD}-0.465\text{CE}+0.442\text{DE}.$$ (5)

Regarding RES production on a banana peel substrate, the highest yield of 113.70 μg/mL was obtained from run no. 31, which consisted of an incubation temperature 30 °C, a pH value 7.0, an inoculum size of 3 mL, an incubation period of 5 days, and a substrate concentration of 10 g/100 mL, followed by run no. 5, which yielded 110.75 μg/mL RES under variables of 30 °C, pH 5.0, inoculum size of 3 mL, incubation period of 10 days, and substrate concentration of 10 g/100 mL. Runs no. 28, 12, and 2, which were conducted at pH 7.0 with variations in the remaining parameters, produced RES yields of 97.40 µg/mL, 91.00 µg/mL, and 83.40 μg/mL, respectively.

Conversely, runs no. 3, 29, 34, and 30 exhibited the lowest RES production (9.05 µg/mL, 9.00 µg/mL, 8.50 µg/mL, and 7.15 μg/mL, respectively), with a 1 g/100 mL substrate concentration and variations in other factors. The remaining experimental runs showed fluctuations in RES yield ranging from 13.95 µg/mL to 75.10 μg/mL, with different levels of optimization variables. Central points of BBD represented by runs 7, 16, 33, 37, 41, and 44 demonstrated RES production values of 44.50 µg/mL, 35.35 µg/mL, 47.2 µg/mL, 34.00 µg/mL, 37.25 µg/mL, and 40.95 μg/mL, respectively (Table 3; Supplementary Figure S2).

Results in Table 3 revealed that the maximum RES yield of 188.80 μg/mL on sugarcane bagasse as a substrate was achieved in run no. 39 under optimized conditions of an incubation temperature 35 °C, a pH value of 5.0, an inoculum size of 3 mL, an incubation period of 10 days, and a substrate concentration of 5.5 g/100 mL, followed by runs 28, 32, and 21 with RES quantities of 176.95 µg/mL, 170.75 µg/mL, and 169.60 μg/mL, respectively, at 35 °C and varied levels of the other factors (Supplementary Figure S3). It is noteworthy that at 35 °C, with variation in other factor levels, RES yields of 131.45 µg/mL, 126.80 µg/mL, 121.55 µg/mL, and 118.30 μg/mL still produced in elevated amounts, as appeared in runs no. 27, 38, 25, and 19, respectively. On the other hand, the lowest RES productivity was obviously in runs no. 26, 3, and 34, with amounts of 7.05 µg/mL, 6.70 µg/mL, and 4.50 μg/mL, respectively, at pH 7.0 and 1 g/100 mL substrate concentration, along with varying conditions of the remaining variables. The remaining optimization runs introduced varied quantities of RES in the range of 114.00–12.10 μg/mL under alternative variable levels. Runs no. 7, 16, 33, 37, 41, and 44, denoted BBD central points, scored RES production of 38.20 µg/mL, 50.20 µg/mL, 42.40 µg/mL, 38.00 µg/mL, 39.00 µg/mL, and 54.90 μg/mL, respectively.

For RES production on the wheat straw substrate, data are presented in Table 3. Run no. 32 produced the best RES yield of 134.85 μg/mL at temperature 35 °C, pH 7.0, inoculum size of 3 mL, incubation period of 15 days, and substrate concentration of 5.5 g/100 mL, followed by run no. 15 with 117.90 μg/mL RES concentration under variable levels including temperature (30 °C), pH (7.0), inoculum size of 3 mL, incubation period of 15 days, and substrate concentration of 10 g/100 mL (Supplementary Figure S4). Runs no. 25, 36, 23, and 28 offered reasonable RES yields of 97.00 µg/mL, 80.30 µg/mL, 75.00 µg/mL, and 73.00 μg/mL, respectively, under varied optimization factor levels. In contrast, the lowest RES production of 9.30 µg/mL, 7.90 µg/mL, 3.25 µg/mL, and 1.70 μg/mL were noted in runs no. 29, 40, 13, and 20, respectively, with different optimization variable levels. Other runs with diverse levels of variables displayed RES concentrations of 68.75–11.30 μg/mL. RES production of 31.20 µg/mL, 41.00 µg/mL, 42.30 µg/mL, 37.15 µg/mL, 35.00 µg/mL, and 40.00 μg/mL were derived from BBD central points’ runs no. 7, 16, 33, 37, 41, and 44, respectively.

Analysis of variance for the RES production quadratic model by the AH26 strain on banana peel substrate is shown in Table 4. Model terms with *p* < 0.05 were considered significant. The F value of the model is 18.55, indicating that the model is significant. A, B, E, D^2^, E^2^, AD, AE, BD, BE, and DE were important model terms in RES production. The “lack of fit F value” of 3.11 was not significant to the pure error. The multiple correlation coefficient, *R* ^2^ = 0.9369, close to one, showed a better association between experimental and predicted values and explained model accuracy with an improved response. Adjusted and predicted *R* ^2^ values were in line with the regression value.

**TABLE 4:** ANOVA for the experimental results of RES production on banana peel substrate by *C. sphaerospermum* AH26.

Source	Sum of squares	Degree of freedom	Mean of squares	F-value	*p*-value
Model	27,571.6	20	1,378.6	18.55	0.000
Linear	18,618.3	5	3,723.7	50.10	0.000
A	383.9	1	383.9	5.17	0.032
B	2,345.0	1	2,345.0	31.55	0.000
C	193.6	1	193.6	2.61	0.119
D	43.6	1	43.6	0.59	0.451
E	15,652.2	1	15,652.2	210.60	0.000
Square	2,547.4	5	509.5	6.85	0.000
A^2^	275.0	1	275.0	3.70	0.066
B^2^	55.2	1	55.2	0.74	0.397
C^2^	149.1	1	149.1	2.01	0.169
D^2^	1,399.9	1	1,399.9	18.83	0.000
E^2^	335.4	1	335.4	4.51	0.044
2-Way interaction	6,405.9	10	640.6	8.62	0.000
AB	185.3	1	185.3	2.49	0.127
AC	61.2	1	61.2	0.82	0.373
AD	2,192.6	1	2,192.6	29.50	0.000
AE	720.9	1	720.9	9.70	0.005
BC	48.7	1	48.7	0.65	0.426
BD	344.1	1	344.1	4.63	0.041
BE	1,045.7	1	1,045.7	14.07	0.001
CD	250.6	1	250.6	3.37	0.078
CE	54.9	1	54.9	0.74	0.398
DE	1,502.0	1	1,502.0	20.21	0.000
Residual	1,858.1	25	74.3	​	​
Lack of fit	1,720.0	20	86.0	3.11	0.106
Pure error	138.1	5	27.6	​	​
Total	29,429.7	45	​	​	​

*R* ^2^, 0.9369; adjusted *R* ^2^, 0.8864; and predicted *R* ^2^, 0.7595.

The Pareto chart and normal probability plot (Supplementary Figure S5a,b) revealed the relationship between optimization parameters and defined the significant variables for RES production on banana peel substrate by AH26 strain. The relationship between parameters and the optimum scale of each factor for RES production was explained by contour plots (Figures 3a–e). The response surface plot (Supplementary Figure S6a–e) was plotted for any two variables while keeping the other variables at their optimal level. Significant interactions were identified by analysis of variance and described as significant model terms that include interactions of incubation temperature with incubation period (AD) and substrate concentration (AE), pH value with incubation period (BD) and substrate concentration (BE), and incubation period with substrate concentration (DE), as shown in the contour plots. The other interactions did not significantly affect RES production.

**FIGURE 3:**
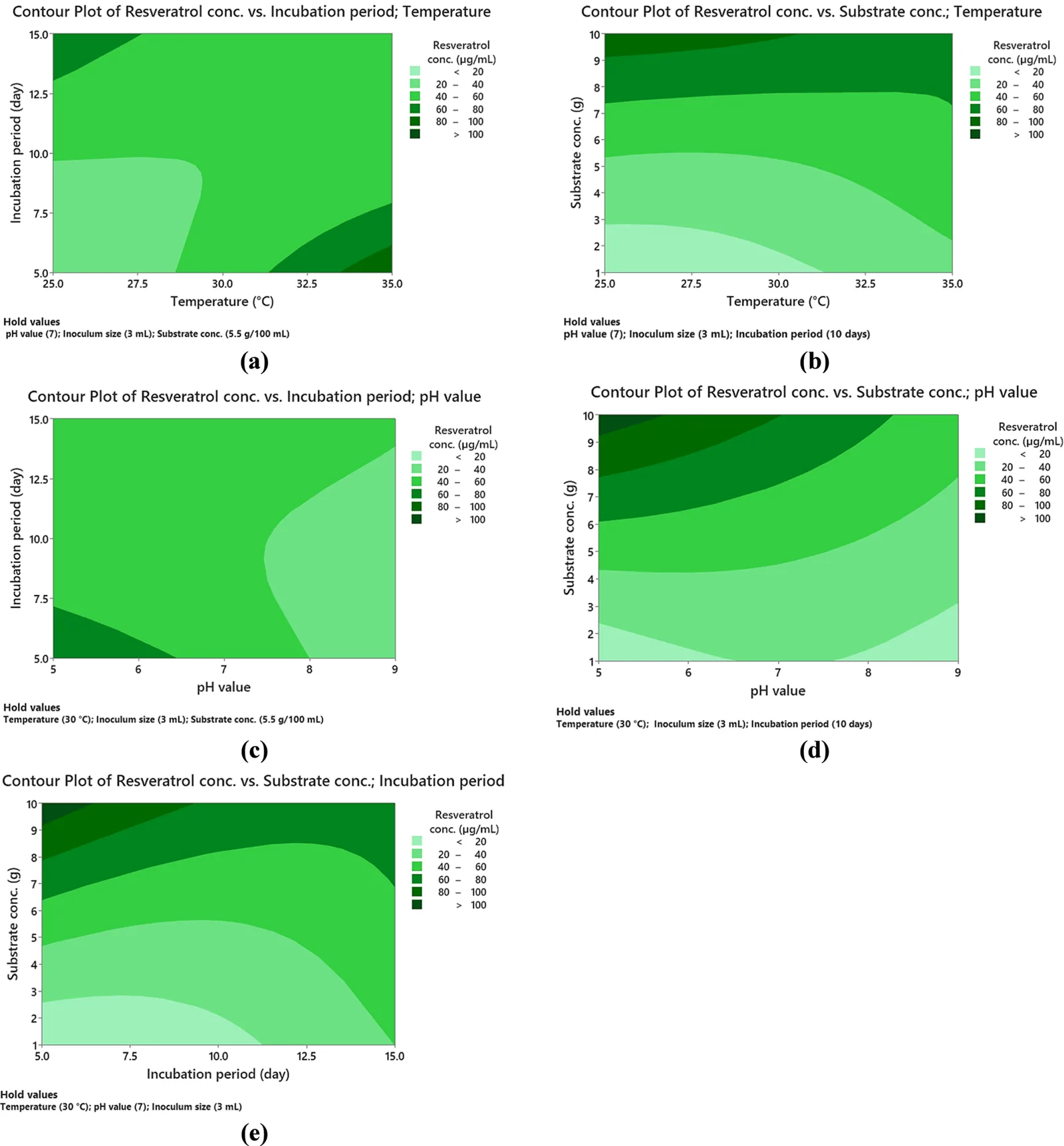
Contour plots of significant interactions between independent variables: **(a)** Incubation temperature versus incubation period; **(b)** incubation temperature versus substrate concentration; **(c)** pH versus incubation period; **(d)** pH versus substrate concentration; **(e)** incubation period versus substrate concentration for RES production on banana peel substrate by *C. sphaerospermum* AH26.

Table 5 shows the quadratic model for RES production on sugarcane bagasse by the AH26 strain, analyzed by ANOVA. The model’s F value of 39.54 proved that the model is significant. In RES production, A, D, E, A^2^, B^2^, D^2^, E^2^, AB, AC, AE, and DE are significant model terms. The “lack of fit F value” of 2.77 was insignificant with respect to the pure error. The preferable correlation between experimental and predicted values was confirmed by the multiple correlation coefficient *R* ^2^ = 0.9694, approximately equal to 1, and explained the model accuracy with an improved response. Moreover, the regression value was consistent with the adjusted and predicted *R* ^2^. Supplementary Figure S7a,b illustrates the relationship between optimization factors and significant parameters by Pareto chart and normal probability plot for the RES yield by fermenting strain AH26 on sugarcane bagasse substrate. The relationship between parameters was explained by contour plots, and the optimum scale of each factor for RES production was determined (Figures 4a–d). Contour and response surface plots (Supplementary Figure S8a–d) and analysis of variance revealed significant interactions. They were described as significant model terms, including interactions between incubation temperature and pH value (AB), inoculum size (AC), substrate concentration (AE), and incubation period and substrate concentration (DE). The remaining interactions exhibited insignificant influence on RES production.

**TABLE 5:** ANOVA for the experimental results of RES production on sugarcane bagasse substrate by *C. sphaerospermum* AH26.

Source	Sum of squares	Degree of freedom	Mean of squares	F-value	*p*-value
Model	97,234	20	4,861.7	39.54	0.000
Linear	55,374	5	11,074.8	90.07	0.000
A	49,645	1	49,645.4	403.76	0.000
B	306	1	305.9	2.49	0.127
C	34	1	34.2	0.28	0.603
D	2,058	1	2,057.8	16.74	0.000
E	3,331	1	3,330.7	27.09	0.000
Square	32,536	5	6,507.3	52.92	0.000
A^2^	19,478	1	19,477.5	158.41	0.000
B^2^	1,250	1	1,249.6	10.16	0.004
C^2^	1	1	1.1	0.01	0.926
D^2^	4,199	1	4,198.5	34.15	0.000
E^2^	3,173	1	3,172.6	25.80	0.000
2-Way interaction	9,323	10	932.3	7.58	0.000
AB	3,094	1	3,094.1	25.16	0.000
AC	1,568	1	1,568.2	12.75	0.001
AD	230	1	229.5	1.87	0.184
AE	724	1	723.6	5.88	0.023
BC	39	1	38.5	0.31	0.581
BD	187	1	187.0	1.52	0.229
BE	364	1	363.9	2.96	0.098
CD	36	1	35.7	0.29	0.595
CE	94	1	93.6	0.76	0.391
DE	2,989	1	2,989.4	24.31	0.000
Residual	3,074	25	123.0	​	​
Lack of fit	2,820	20	141.0	2.77	0.131
Pure error	254	5	50.8	​	​
Total	100,308	45	​	​	​

*R* ^2^, 0.9694; adjusted *R* ^2^, 0.9448; and predicted *R* ^2^, 0.8839.

**FIGURE 4:**
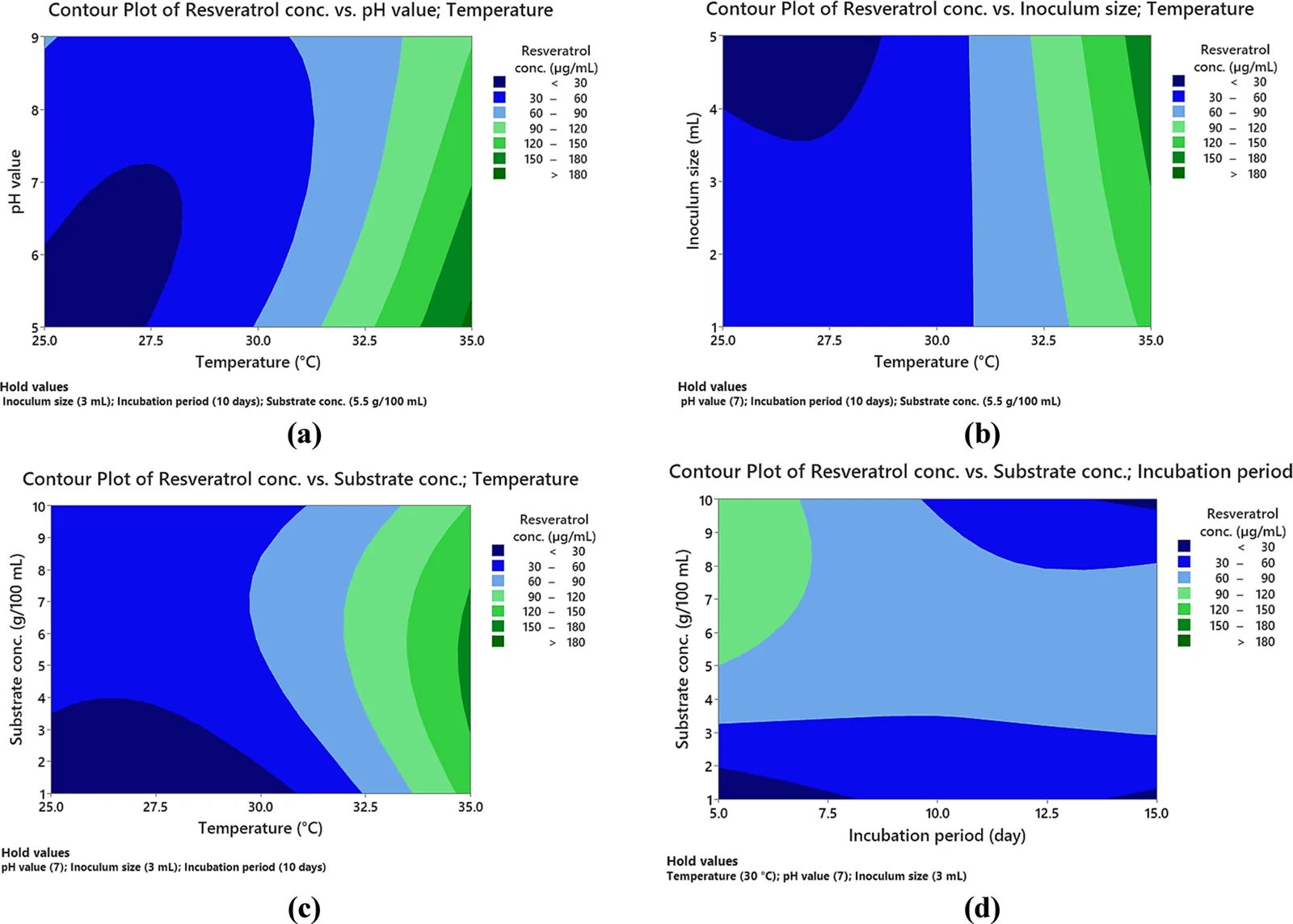
Contour plots of significant interactions between independent variables: **(a)** incubation temperature with pH value, **(b)** incubation temperature with inoculum size, **(c)** incubation temperature with substrate concentration, and **(d)** incubation period with substrate concentration for RES production on sugarcane bagasse substrate by *C. sphaerospermum* AH26.

Table 6 shows the quadratic model for RES production on wheat straw using the AH26 strain analyzed by ANOVA. The model’s F value of 13.49 proved that the model is significant. In RES production, the important model terms are A, D, E, A^2^, B^2^, C^2^, D^2^, AD, AE, and BE. However, the “lack of fit F value” of 8.91 showed a significant concern about the pure error. The multiple correlation coefficient (*R* ^2^ = 0.9152) near 1 indicated a preferable interconnection between experimental and predicted values (adjusted and predicted *R* ^2^ values were 0.8473 and 0.6667, respectively). The Pareto chart and normal probability plot, shown in Supplementary Figure S9a,b showed the relationship between the optimization variable and identified the significant parameters for RES concentration determined by submerged fermentation of AH26 strain on wheat substrate. Contour plots described the relationship between parameters and defined the optimum scale of each factor for RES production (Figures 5a–c). Response surface plots (Supplementary Figure S10a–c) showed significant interactions from the analysis of variance. Significant model terms included interactions between incubation temperature and incubation period (AD), substrate concentration (AE), and pH value and substrate concentration (BE). Other interactions did not significantly affect RES production.

**TABLE 6:** ANOVA for the experimental results of RES production on wheat straw substrate by *C. sphaerospermum* AH26.

Source	Sum of squares	Degree of freedom	Mean of squares	F-value	*p*-value
Model	34,457.2	20	1,722.86	13.49	0.000
Linear	19,145.6	5	3,829.12	29.98	0.000
A	5,648.3	1	5,648.27	44.22	0.000
B	9.2	1	9.15	0.07	0.791
C	506.8	1	506.81	3.97	0.057
D	4,875.5	1	4,875.53	38.17	0.000
E	8,105.9	1	8,105.85	63.46	0.000
Square	9,946.2	5	1,989.25	15.57	0.000
A^2^	1,373.2	1	1,373.24	10.75	0.003
B^2^	1,254.9	1	1,254.85	9.82	0.004
C^2^	1,434.4	1	1,434.39	11.23	0.003
D^2^	3,225.8	1	3,225.82	25.26	0.000
E^2^	39.0	1	39.03	0.31	0.585
2-Way interaction	5,365.3	10	536.53	4.20	0.002
AB	374.4	1	374.42	2.93	0.099
AC	0.0	1	0.03	0.00	0.988
AD	1,008.1	1	1,008.06	7.89	0.009
AE	2,387.8	1	2,387.79	18.69	0.000
BC	11.2	1	11.22	0.09	0.769
BD	11.6	1	11.56	0.09	0.766
BE	1,085.7	1	1,085.70	8.50	0.007
CD	21.4	1	21.39	0.17	0.686
CE	70.1	1	70.14	0.55	0.466
DE	395.0	1	395.02	3.09	0.091
Residual	3,193.1	25	127.72	13.49	0.000
Lack of fit	3,106.0	20	155.30	8.91	0.012
Pure error	87.1	5	17.43	​	​
Total	37,650.3	45	​	​	​

*R* ^2^, 0.9152; adjusted *R* ^2^, 0.8473; and predicted *R* ^2^, 0.6667.

**FIGURE 5:**
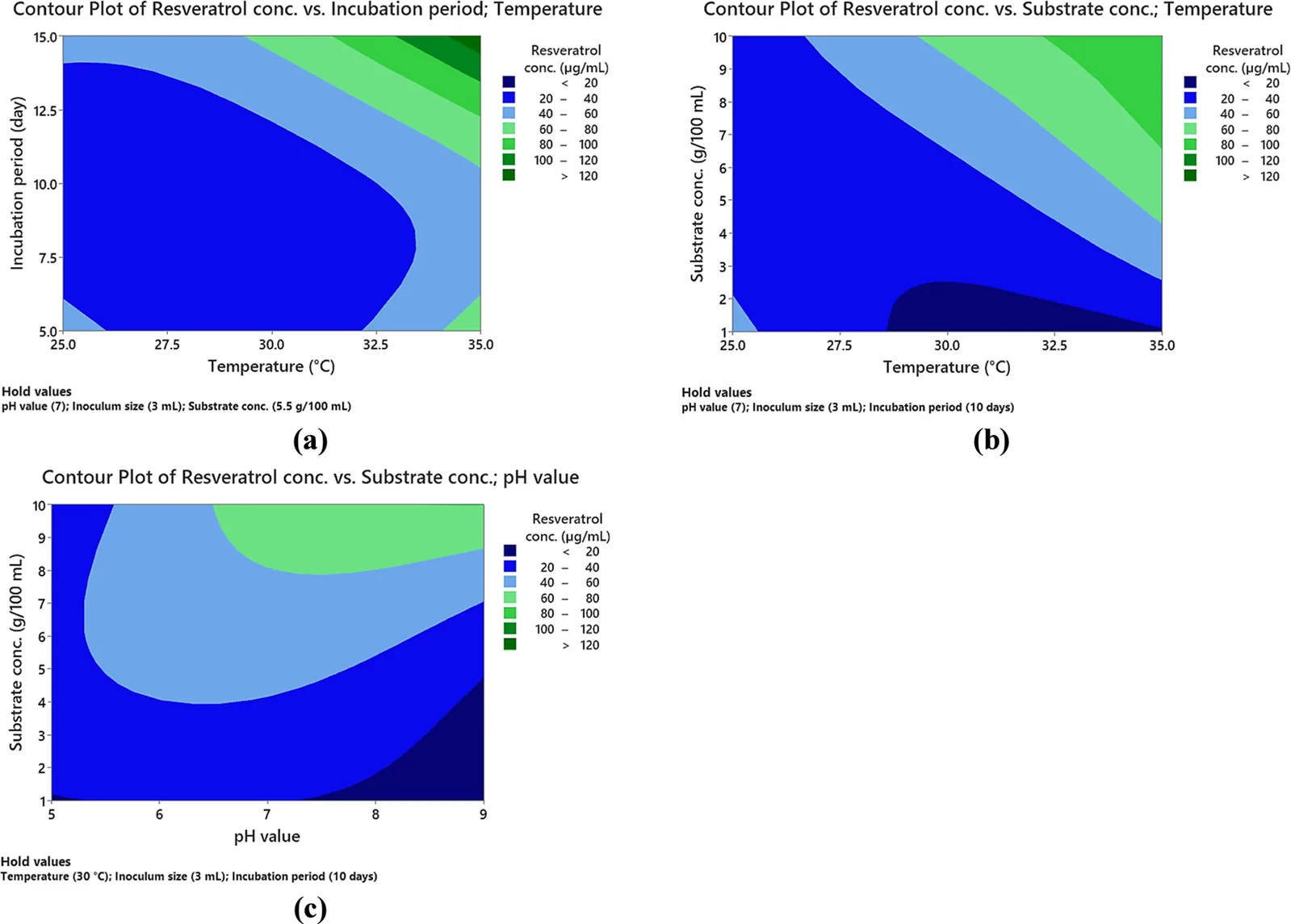
Contour plots of the significant interactions of independent variables: **(a)** incubation temperature and period; **(b)** incubation temperature and substrate concentration; **(c)** pH value and substrate concentration for RES production from wheat straw substrate by *C. sphaerospermum* AH26.

### 3.5 Correlation between RES productivity and enzymatic activities on waste substrate

The results in Supplementary Table S1 and Figure 6 present amylase, cellulase, and pectinase activities on banana peel substrate fermented by AH26 in relation to RES concentration in BBD-46 runs with varied factorial levels. Data showed agreement between RES concentration and enzyme activity, where amylase production scored high activity, followed by cellulase and pectinase. At high-RES productivity (113.70 μg/mL) in run no. 31, amylase, cellulase, and pectinase activities were 15.02 U/mL, 12.76 U/mL, and 10.28 U/mL, respectively, while at lowest concentration of RES (7.15 μg/mL) in run no. 30, low activities of 2.76 U/mL, 0.95 U/mL, and 0.88 U/mL were reported for amylase, cellulase, and pectinase, respectively. Pearson’s correlation of RES production using RSM-BBD (46 runs) and extracellular amylase, cellulase, and pectinase activities on banana peel substrate by AH26 submerged fermentation revealed significant positive correlations between tested values at the 0.01 level of significance (Table 7).

**FIGURE 6:**
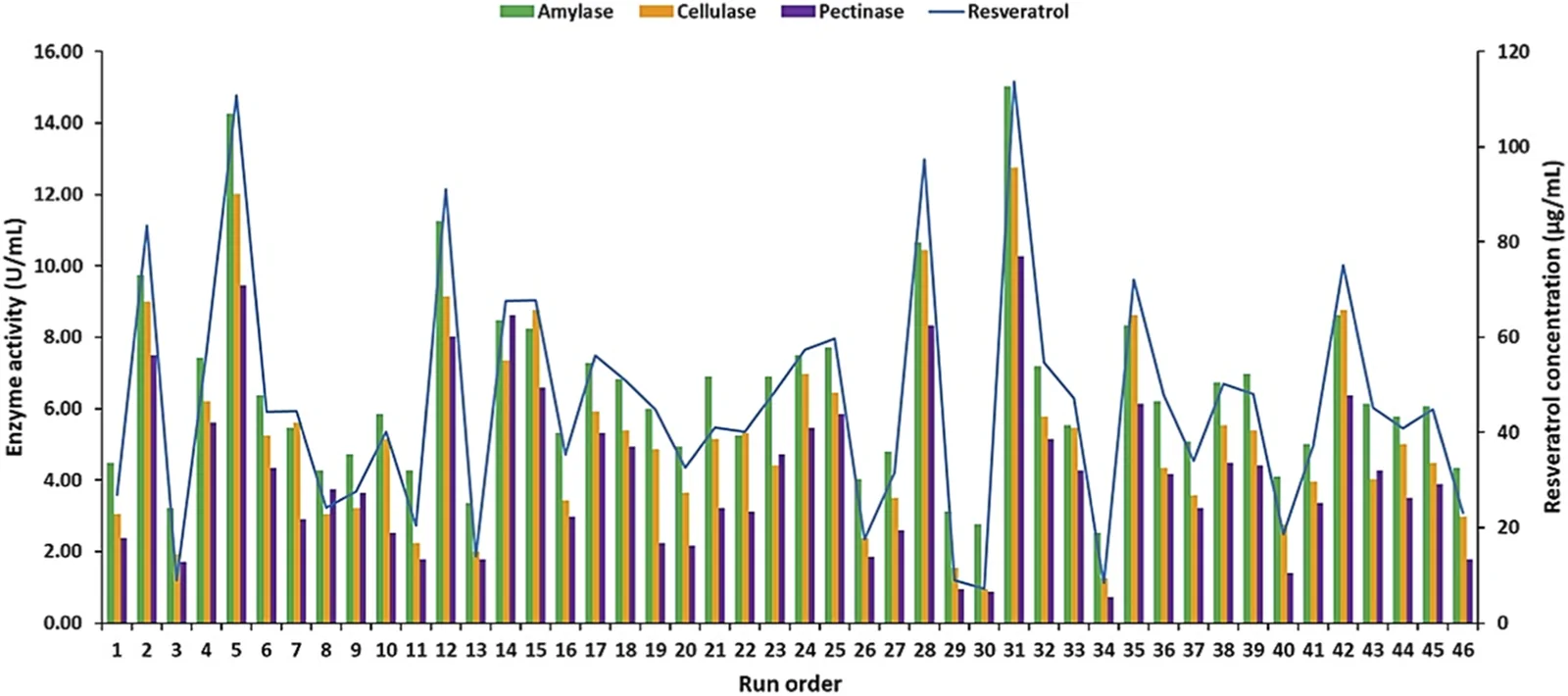
RES productivity using RSM-BBD and enzymatic activities on banana peel substrate by *C. sphaerospermum* AH26.

**TABLE 7:** Pearson’s correlation coefficients of RES production using RSM-BBD and enzyme activities on banana peel substrate by *C. sphaerospermum* AH26.

Parameter	RES	Amylase	Cellulase	Pectinase
RES	1	0.980**	0.983**	0.958**
Amylase	0.980**	1	0.965**	0.946**
Cellulase	0.983**	0.965**	1	0.938**
Pectinase	0.958**	0.946**	0.938**	1

Data are presented for N = 46.

** Correlation is significant at the 0.01 level (2-tailed).

It was notable that no enzymatic activities were detected for amylase, cellulase, pectinase, and xylanase on sugarcane bagasse substrate subjected to SmF by AH26 under different BBD parameters. Data in Supplementary Table S2 and Figure 7 revealed a directly proportional relationship between RES concentration and enzyme activity on wheat straw fermented by the AH26 strain, with cellulase achieving the highest activity. High RES production of 134.85 μg/mL in run no. 32 was accompanied by 16.52 U/mL, 8.25 U/mL, and 6.37 U/mL activity attained by cellulase, xylanase, and amylase, respectively. Meanwhile, run no. 20 showed low cellulase (1.85 U/mL), xylanase (0.73 U/mL), and amylase (0.88 U/mL) activities and exhibited the lowest RES yield (1.70 μg/mL). Using RSM-BBD (46 runs), RES production in submerged fermentation by AH26, Pearson’s correlation of RES concentration and amylase, cellulase, and xylanase activities on wheat straw substrate exhibited significant positive correlations between tested values at the 0.01 level of significance (Table 8).

**FIGURE 7:**
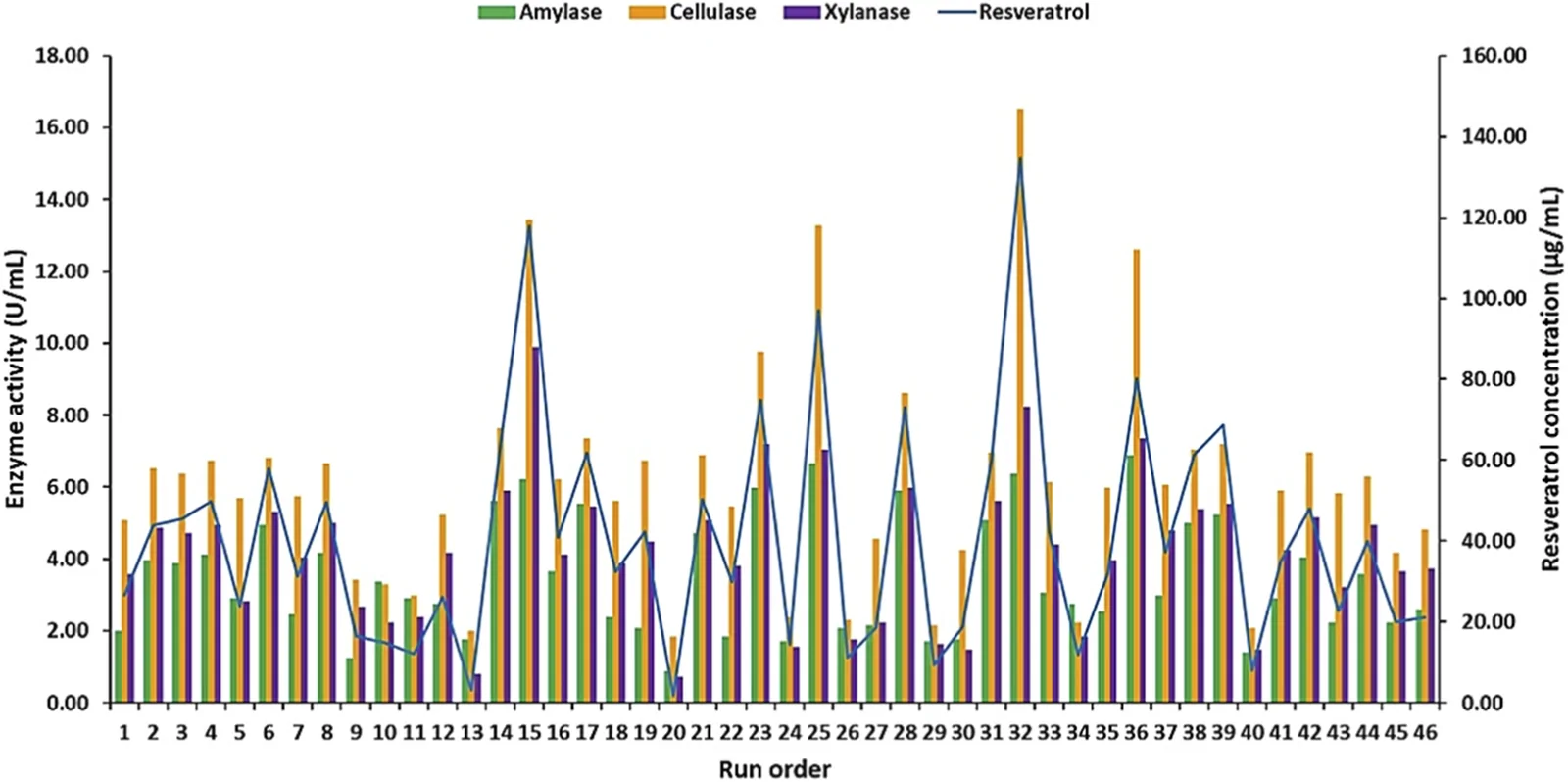
RES productivity using RSM-BBD and enzymatic activities on wheat straw substrate by *C. sphaerospermum* AH26.

**TABLE 8:** Pearson’s correlation coefficients of RES production using RSM-BBD and enzyme activities on wheat straw substrate by *C. sphaerospermum* AH26.

Parameter	RES	Amylase	Cellulase	Xylanase
RES	1	0.901**	0.965**	0.949**
Amylase	0.901**	1	0.847**	0.880**
Cellulase	0.965**	0.847**	1	0.931**
Xylanase	0.949**	0.880**	0.931**	1

Data are presented for N = 46.

** Correlation is significant at the 0.01 level (two-tailed).

## 4 Discussion

Endophytic fungi act as bio-factories for RES production, due to their inherent capacity for synthesizing plant secondary metabolites as a result of long-term coexistence with host plants. Numerous grapevine-derived fungal endophytes have been reported to produce RES, including *Alternaria* sp., *Penicillium* sp., *Aspergillus niger*, *Xylaria* sp., *Quambalaria cyanescens*, and *Fusarium equiseti* (Abo-Kadoum et al., 2022). Synthetic and semi-synthetic media such as Czapek Dox broth (CDB) and potato dextrose broth (PDB) are frequently utilized for screening and optimization of RES production by myco-endophytes (Dwibedi and Saxena, 2019b; Roat and Saraf, 2020; Abo-Kadoum et al., 2022; Meshram et al., 2022; Al Mousa et al., 2026). However, despite their usefulness, these media lack complexity, nutrient diversity, and economic sustainability. On the other hand, agricultural wastes represent inexpensive and sustainable carbon substrates to produce value-added products such as RES (Sayyed et al., 2021). Utilization of such materials for RES production remains limited. Therefore, the present study was conducted to evaluate agricultural waste such as banana peel, sugarcane bagasse, and wheat straw as potential substrates for production and optimization of RES, along with the associated hydrolytic enzymatic activities using grapevine-derived fungus *C. sphaerospermum*.

The genus *Cladosporium* has not been reported for RES production. However, Aleynova et al. (2021) demonstrated that *Cladosporium* can induce stilbene accumulation in *Vitis amurensis* cells during co-cultivation, highlighting its role as an elicitor rather than an RES producer. The primary screening of *C. sphaerospermum* exhibited its ability to produce 42.3 μg/mL RES after 7 days of cultivation on PDB medium. This amount is substantially higher than previously reported *V. vinifera*-derived strains cultivated on PDB medium, *F. solani* (31.3 μg/mL) and *X. psidii* (35.43 μg/mL) (Dwibedi and Saxena, 2019b), *A. stellifer* (0.288 μg/mL) (Roat and Saraf, 2020), and *Quambalaria cyanescens* (40 μg/mL) on CDB (Meshram et al., 2022). Our finding introduces *C. sphaerospermum* as an efficient candidate for commercial RES production. *C. sphaerospermum* exhibited RES production capability upon cultivation on banana peel, wheat straw, and sugarcane bagasse in comparable amounts to that obtained on PDB medium, 41.78 µg/mL, 39.59 µg/mL, and 40.33 µg/mL, respectively.

RES production by mycoendophytes is largely influenced by culture conditions and nutrient availability. Optimization of these factors can maximize RES productivity (Shi et al., 2012; Dwibedi et al., 2021). RSM statistical analysis offers an ideal approach that enables identification of the most significant variables that affect RES production through linear or quadratic models, as well as variable interactions, and determining their optimum levels for the desired response (Dwibedi et al., 2021). In this regard, five common variables were tested: temperature, pH, inoculum size, incubation period, and substrate concentration to optimize RES productivity upon agricultural waste cultivation. ANOVA revealed that most of the tested variables significantly influence RES productivity in linear and/or quadratic terms. Among the factors investigated, temperature and substrate concentration significantly affected RES production across all tested wastes. The interaction of temperature with other parameters, including incubation period, pH, and substrate concentration, significantly influenced RES productivity on banana peel, sugarcane bagasse, and wheat straw, respectively. This finding highlights the synergistic effect of these interactions and emphasizes the impact of the RSM approach for parameter optimization to maximize RES yield (Shi et al., 2012; Lu et al., 2016; Dwibedi et al., 2021).

Previous studies have reported that accumulations of RES by endophytic fungi begin from day one and increase proportionally with biomass progression under moderate temperature, neutral pH, and sufficient substrate availability. These conditions maintain physiological stability and support biomass formation, which requires stable production of RES (Shi et al., 2012; Dwibedi et al., 2021; Abo-Kadoum et al., 2022). Consistent with the previous reports, the highest RES yield of 113.70 μg/mL was obtained with 10 g of banana peel and a 3 mL inoculum size after 5 days at 30 °C and pH 7. However, only 5.5 g of sugarcane bagasse and wheat straw with 3 mL of inoculum was required at an elevated temperature of 35 °C and a slightly low pH of 5 to reach the top RES levels of 188.80 µg/mL and 134.85 µg/mL after 10 days and 15 days, respectively.

The variation in cultivation conditions may be due to the chemical composition and nutrient availability of different wastes (Gupta and Jana, 2018; El-Katony et al., 2020). Banana peel contains a high fraction of simple fermentable compounds that facilitate fast growth under moderate conditions and enable RES accumulation within a short period (Pereira et al., 2021a). Therefore, higher banana peel concentrations than other wastes are reasonable. On the other hand, wheat straw and sugarcane bagasse are rich in lignocellulosic substances and complex polymers, which require stress conditions to elicit enzymatic activity, thereby delaying substrate utilization and subsequently RES accumulation. Therefore, interaction of temperature with pH and substrate concentration is crucial to fine-tune fungal response toward agricultural waste substrates. It has been reported that lignocellulose-degrading fungi cultivated on sugarcane bagasse exhibited optimal degrading capacity at slightly acidic pH and elevated temperatures of 30 °C–40 °C (Almeida et al., 2022; Benatti and Polizeli, 2023). The highest level of RES on all agricultural wastes was obtained with the center point of inoculum size (3 mL), underscoring the inhibitory effect of either low or excessive inoculum volume that undermines fungal growth and subsequently decreases RES yield (Shi et al., 2012). Collectively, our findings reflect a substrate-dependent adaptation strategy adopted by *C. sphaerospermum* under different agricultural wastes to maximize RES yield.

Hydrolytic enzyme activity is a prerequisite for fungal cultivation on agricultural wastes (Alananbeh et al., 2024). It liberates fermentable sugars that are directed toward the shikimate pathway and subsequently the phenylpropanoid pathway for RES synthesis (Kobayashi et al., 2021). *C. sphaerospermum* showed extracellular enzymatic activity for all tested enzymes, including amylase, cellulase, pectinase, and xylanase, upon cultivation on banana peel and wheat straw, except for pectinase. The lack of enzymatic activity with sugarcane bagasse may likely be due to high lignin content that hampers enzymatic accessibility, especially in the absence of pretreatment, and constitutes a stress condition that may favor RES production (Rashid et al., 2020). Also, the presence of residual soluble sugars impedes enzyme production and prioritizes the consumption of easily metabolized sugars through carbon catabolite repression (CCR) (Antonieto et al., 2014). The correlation between RES yield and enzymatic activity on agricultural wastes was addressed using BBD-46 runs with varied factorial levels. As expected, RES production rate by *C. sphaerospermum* was proportionally increased concomitant with the activity of amylase, cellulase, and pectinase under both banana peel and wheat straw, with Pearson’s correlation coefficient of approximately 1. Cellulose is the major carbohydrate constituent in banana peel followed by starches and pectin (Gopi et al., 2014). However, it is always found associated with lignin fibers that hamper enzymatic access (Pereira et al., 2021b; Mishra et al., 2023). On the other hand, starch can easily be metabolized by amylase (Li et al., 2018). Therefore, banana peel was a more popular substrate for amylase production and optimization by fungi. Accordingly, the enzymatic activity pattern of *C. sphaerospermum* showed superior performance for amylase, followed by cellulase and pectinase. This pattern was altered upon wheat straw cultivation due to its lignocellulosic composition that elicits cellulase activity rather than amylase or pectinase (Pensupa et al., 2013).

## 5 Conclusion

In this study, we isolated and identified the grape-derived endophytic fungus *C. sphaerospermum* AH26 as a novel efficient strain for production of RES; our strain exhibited great potential to produce RES on different agricultural wastes. Culture conditions were modified, and the yield of RES was optimized by RSM. The positive correlation between hydrolytic enzyme activities and RES production will facilitate the investigation of whether the overexpression of agricultural waste-degrading enzymes might directly enhance RES yield by *C. sphaerospermum*. To the best of our knowledge, this is the first report that addresses agricultural wastes as a potential low-cost substrate for production and optimization of RES by mycoendophytes.

## Data Availability

The datasets presented in this study can be found in online repositories. The names of the repository/repositories and accession number(s) can be found below: https://www.ncbi.nlm.nih.gov/, PX952784.
