# Supplementary material for: Leveraging agricultural waste manipulation using a novel resveratrol-producing endophytic fungus, Cladosporium sphaerospermum AH26: optimization and enzymatic investigation
Source: Front Bioeng Biotechnol. 2026 Sep 16;14:1933402. doi: 10.3389/fbioe.2026.1933402 (PMC13623705; doi:10.3389/fbioe.2026.1933402)
Supplement: Supplementary file 1 [file Supplementaryfile1.docx]

**Supplementary**

Figure S1. HPLC chromatogram of RES standard (0.15 mg/mL).

Figure S2. HPLC chromatograms of highest RES production on banana peel.

Figure S3. HPLC chromatograms of highest RES production on sugarcane bagasse.

Figure S4. HPLC chromatograms of highest RES production on wheat straw.

|  | |  |
| --- | --- | --- |
| **(a)** | |  |
|  | |  |
| **(b)** | |  |
| **Figure S5. (a)** Pareto chart and **(b)** normal probability plot, examined in BBD design, showing the effects of the investigated factors on RES production by submerged fermentation of banana peel by *C. sphaerospermum* AH26. | |  |
|  |  | |
| **(a)** | **(b)** | |
|  |  | |
| **(c)** | **(d)** | |
|  |  | |
| **(e)** |  | |
| **Figure S6.** Surface plots showing significant interactions between independent variables: **(a)** incubation temperature with incubation period; **(b)** incubation temperature with substrate concentration; **(c)** pH value with incubation period; **(d)** pH value with substrate concentration; **(e)** incubation period with substrate concentration for RES production on banana peel substrate by *C. sphaerospermum* AH26. | | |
|  | |  |
| **(a)** | |  |
|  | |  |
| **(b)** | |  |
| **Figure S7. (a)** Pareto chart and **(b)** normal probability plot, examined in BBD design, showing the effects of the investigated factors on RES production by submerged fermentation of sugarcane bagasse by *C. sphaerospermum* AH26. | |  |

|  |  |
| --- | --- |
| **(a)** | **(b)** |
|  |  |
| **(c)** | **(d)** |
| **Figure S8.** Surface plots showing significant interactions between independent variables: **(a)** incubation temperature with pH value; **(b)** incubation temperature with inoculum size; **(c)** incubation temperature with substrate concentration; **(d)** incubation period with substrate concentration for RES production on sugarcane bagasse substrate by *C. sphaerospermum* AH26. | |

**(a)**

**(b)**

**Figure S9. (a)** Pareto chart and **(b)** normal probability plot, examined in BBD design, showing the effects of the investigated factors on RES production by submerged fermentation of wheat straw by *C. sphaerospermum* AH26.

|  |  |
| --- | --- |
| **(a)** | **(b)** |
|  |  |
| **(c)** |  |
| **Figure S10.** Surface plots showing significant interactions between independent variables: **(a)** incubation temperature with period; **(b)** incubation temperature with substrate concentration; **(c)** pH value with substrate concentration for RES production on wheat straw substrate by *C. sphaerospermum* AH26. | |

**Table** **S1.** Enzymatic activity with experimental RES production (µg/mL) on banana peel substrate by *C. sphaerospermum* AH26.

| **Run Order** | **Variables** | | | | | **RES concentration**  **(µg/mL)** | **Enzyme activity (U/mL)** | | |
| --- | --- | --- | --- | --- | --- | --- | --- | --- | --- |
|  | **A** | **B** | **C** | **D** | **E** |  | **Amylase** | **Cellulase** | **Pectinase** |
| 1 | 25 | 9 | 3 | 10 | 5.5 | 26.90 | 4.49 | 3.06 | 2.38 |
| 2 | 30 | 7 | 5 | 10 | 10 | 83.40 | 9.75 | 9.00 | 7.49 |
| 3 | 25 | 7 | 3 | 10 | 1 | 9.05 | 3.21 | 1.93 | 1.70 |
| 4 | 30 | 7 | 5 | 15 | 5.5 | 57.00 | 7.42 | 6.22 | 5.61 |
| 5 | 30 | 5 | 3 | 10 | 10 | 110.75 | 14.26 | 12.01 | 9.45 |
| 6 | 30 | 9 | 3 | 15 | 5.5 | 44.30 | 6.37 | 5.24 | 4.34 |
| 7 | 30 | 7 | 3 | 10 | 5.5 | 44.50 | 5.46 | 5.61 | 2.91 |
| 8 | 25 | 7 | 3 | 5 | 5.5 | 24.15 | 4.26 | 3.06 | 3.73 |
| 9 | 30 | 7 | 5 | 5 | 5.5 | 27.55 | 4.71 | 3.21 | 3.66 |
| 10 | 30 | 5 | 5 | 10 | 5.5 | 40.15 | 5.84 | 5.16 | 2.53 |
| 11 | 25 | 7 | 5 | 10 | 5.5 | 20.40 | 4.26 | 2.23 | 1.78 |
| 12 | 25 | 7 | 3 | 10 | 10 | 91.00 | 11.25 | 9.15 | 8.02 |
| 13 | 30 | 9 | 5 | 10 | 5.5 | 13.95 | 3.36 | 2.00 | 1.78 |
| 14 | 30 | 7 | 1 | 10 | 10 | 67.65 | 8.47 | 7.34 | 8.62 |
| 15 | 30 | 7 | 3 | 15 | 10 | 67.85 | 8.25 | 8.77 | 6.59 |
| 16 | 30 | 7 | 3 | 10 | 5.5 | 35.35 | 5.31 | 3.43 | 2.98 |
| 17 | 30 | 5 | 3 | 15 | 5.5 | 56.10 | 7.27 | 5.91 | 5.31 |
| 18 | 30 | 7 | 1 | 5 | 5.5 | 50.85 | 6.82 | 5.39 | 4.94 |
| 19 | 35 | 9 | 3 | 10 | 5.5 | 44.85 | 5.99 | 4.86 | 2.23 |
| 20 | 30 | 9 | 1 | 10 | 5.5 | 32.60 | 4.94 | 3.66 | 2.15 |
| 21 | 35 | 7 | 5 | 10 | 5.5 | 41.00 | 6.91 | 5.16 | 3.21 |
| 22 | 30 | 7 | 3 | 15 | 1 | 40.16 | 5.24 | 5.31 | 3.13 |
| 23 | 30 | 7 | 1 | 15 | 5.5 | 48.64 | 6.89 | 4.41 | 4.71 |
| 24 | 25 | 5 | 3 | 10 | 5.5 | 57.30 | 7.49 | 6.97 | 5.46 |
| 25 | 35 | 7 | 3 | 10 | 10 | 59.80 | 7.72 | 6.44 | 5.84 |
| 26 | 30 | 7 | 1 | 10 | 1 | 17.65 | 4.03 | 2.38 | 1.85 |
| 27 | 35 | 7 | 3 | 10 | 1 | 31.55 | 4.79 | 3.51 | 2.61 |
| 28 | 35 | 7 | 3 | 5 | 5.5 | 97.40 | 10.65 | 10.43 | 8.32 |
| 29 | 30 | 9 | 3 | 10 | 1 | 9.00 | 3.13 | 1.55 | 0.95 |
| 30 | 30 | 5 | 3 | 10 | 1 | 7.15 | 2.76 | 0.95 | 0.88 |
| 31 | 30 | 7 | 3 | 5 | 10 | 113.70 | 15.02 | 12.76 | 10.28 |
| 32 | 35 | 7 | 3 | 15 | 5.5 | 54.70 | 7.19 | 5.76 | 5.16 |
| 33 | 30 | 7 | 3 | 10 | 5.5 | 47.20 | 5.54 | 5.46 | 4.26 |
| 34 | 30 | 7 | 3 | 5 | 1 | 8.50 | 2.53 | 1.25 | 0.73 |
| 35 | 30 | 5 | 3 | 5 | 5.5 | 72.10 | 8.32 | 8.62 | 6.14 |
| 36 | 30 | 9 | 3 | 10 | 10 | 47.93 | 6.22 | 4.34 | 4.18 |
| 37 | 30 | 7 | 3 | 10 | 5.5 | 34.00 | 5.09 | 3.58 | 3.21 |
| 38 | 35 | 7 | 1 | 10 | 5.5 | 50.20 | 6.74 | 5.54 | 4.49 |
| 39 | 35 | 5 | 3 | 10 | 5.5 | 48.03 | 6.97 | 5.39 | 4.41 |
| 40 | 30 | 7 | 5 | 10 | 1 | 18.58 | 4.11 | 2.76 | 1.40 |
| 41 | 30 | 7 | 3 | 10 | 5.5 | 37.25 | 5.01 | 3.96 | 3.36 |
| 42 | 25 | 7 | 3 | 15 | 5.5 | 75.10 | 8.62 | 8.77 | 6.37 |
| 43 | 25 | 7 | 1 | 10 | 5.5 | 45.25 | 6.14 | 4.03 | 4.26 |
| 44 | 30 | 7 | 3 | 10 | 5.5 | 40.95 | 5.76 | 5.01 | 3.51 |
| 45 | 30 | 5 | 1 | 10 | 5.5 | 44.85 | 6.07 | 4.49 | 3.88 |
| 46 | 30 | 9 | 3 | 5 | 5.5 | 23.20 | 4.34 | 2.98 | 1.78 |

**Table** **S2.** Enzymatic activity with experimental RES production (µg/mL) on wheat straw substrate by *C. sphaerospermum* AH26.

| **Run Order** | **Variables** | | | | | **RES concentration**  **(µg/mL)** | **Enzyme activity (U/mL)** | | |
| --- | --- | --- | --- | --- | --- | --- | --- | --- | --- |
|  | **A** | **B** | **C** | **D** | **E** |  | **Amylase** | **Cellulase** | **Pectinase** |
| 1 | 25 | 9 | 3 | 10 | 5.5 | 26.65 | 2.00 | 5.09 | 3.58 |
| 2 | 30 | 7 | 5 | 10 | 10 | 43.85 | 3.96 | 6.52 | 4.86 |
| 3 | 25 | 7 | 3 | 10 | 1 | 45.45 | 3.88 | 6.37 | 4.71 |
| 4 | 30 | 7 | 5 | 15 | 5.5 | 49.85 | 4.11 | 6.74 | 4.94 |
| 5 | 30 | 5 | 3 | 10 | 10 | 23.85 | 2.91 | 5.69 | 2.83 |
| 6 | 30 | 9 | 3 | 15 | 5.5 | 57.90 | 4.94 | 6.82 | 5.31 |
| 7 | 30 | 7 | 3 | 10 | 5.5 | 31.20 | 2.45 | 5.76 | 4.03 |
| 8 | 25 | 7 | 3 | 5 | 5.5 | 49.65 | 4.18 | 6.67 | 5.01 |
| 9 | 30 | 7 | 5 | 5 | 5.5 | 16.40 | 1.25 | 3.43 | 2.68 |
| 10 | 30 | 5 | 5 | 10 | 5.5 | 14.85 | 3.36 | 3.28 | 2.23 |
| 11 | 25 | 7 | 5 | 10 | 5.5 | 12.05 | 2.91 | 2.98 | 2.38 |
| 12 | 25 | 7 | 3 | 10 | 10 | 26.20 | 2.76 | 5.24 | 4.18 |
| 13 | 30 | 9 | 5 | 10 | 5.5 | 3.25 | 1.78 | 2.00 | 0.80 |
| 14 | 30 | 7 | 1 | 10 | 10 | 64.00 | 5.61 | 7.64 | 5.91 |
| 15 | 30 | 7 | 3 | 15 | 10 | 117.90 | 6.22 | 13.44 | 9.90 |
| 16 | 30 | 7 | 3 | 10 | 5.5 | 41.00 | 3.66 | 6.22 | 4.11 |
| 17 | 30 | 5 | 3 | 15 | 5.5 | 61.90 | 5.54 | 7.34 | 5.46 |
| 18 | 30 | 7 | 1 | 5 | 5.5 | 32.30 | 2.38 | 5.61 | 3.88 |
| 19 | 35 | 9 | 3 | 10 | 5.5 | 42.20 | 2.08 | 6.74 | 4.49 |
| 20 | 30 | 9 | 1 | 10 | 5.5 | 1.70 | 0.88 | 1.85 | 0.73 |
| 21 | 35 | 7 | 5 | 10 | 5.5 | 50.25 | 4.71 | 6.89 | 5.09 |
| 22 | 30 | 7 | 3 | 15 | 1 | 29.95 | 1.85 | 5.46 | 3.81 |
| 23 | 30 | 7 | 1 | 15 | 5.5 | 75.00 | 5.99 | 9.75 | 7.19 |
| 24 | 25 | 5 | 3 | 10 | 5.5 | 14.50 | 1.70 | 2.38 | 1.55 |
| 25 | 35 | 7 | 3 | 10 | 10 | 97.00 | 6.67 | 13.29 | 7.04 |
| 26 | 30 | 7 | 1 | 10 | 1 | 11.30 | 2.08 | 2.30 | 1.78 |
| 27 | 35 | 7 | 3 | 10 | 1 | 18.52 | 2.15 | 4.56 | 2.23 |
| 28 | 35 | 7 | 3 | 5 | 5.5 | 73.00 | 5.91 | 8.62 | 5.99 |
| 29 | 30 | 9 | 3 | 10 | 1 | 9.30 | 1.70 | 2.15 | 1.63 |
| 30 | 30 | 5 | 3 | 10 | 1 | 18.75 | 1.78 | 4.26 | 1.48 |
| 31 | 30 | 7 | 3 | 5 | 10 | 60.00 | 5.09 | 6.97 | 5.61 |
| 32 | 35 | 7 | 3 | 15 | 5.5 | 134.85 | 6.37 | 16.52 | 8.25 |
| 33 | 30 | 7 | 3 | 10 | 5.5 | 42.30 | 3.06 | 6.14 | 4.41 |
| 34 | 30 | 7 | 3 | 5 | 1 | 11.80 | 2.76 | 2.23 | 1.85 |
| 35 | 30 | 5 | 3 | 5 | 5.5 | 31.85 | 2.53 | 5.99 | 3.96 |
| 36 | 30 | 9 | 3 | 10 | 10 | 80.30 | 6.89 | 12.61 | 7.34 |
| 37 | 30 | 7 | 3 | 10 | 5.5 | 37.15 | 2.98 | 6.07 | 4.79 |
| 38 | 35 | 7 | 1 | 10 | 5.5 | 61.35 | 5.01 | 7.04 | 5.39 |
| 39 | 35 | 5 | 3 | 10 | 5.5 | 68.75 | 5.24 | 7.19 | 5.54 |
| 40 | 30 | 7 | 5 | 10 | 1 | 7.90 | 1.40 | 2.08 | 1.48 |
| 41 | 30 | 7 | 3 | 10 | 5.5 | 35.00 | 2.91 | 5.91 | 4.26 |
| 42 | 25 | 7 | 3 | 15 | 5.5 | 48.00 | 4.03 | 6.97 | 5.16 |
| 43 | 25 | 7 | 1 | 10 | 5.5 | 22.80 | 2.23 | 5.84 | 3.21 |
| 44 | 30 | 7 | 3 | 10 | 5.5 | 40.00 | 3.58 | 6.29 | 4.94 |
| 45 | 30 | 5 | 1 | 10 | 5.5 | 20.00 | 2.23 | 4.18 | 3.66 |
| 46 | 30 | 9 | 3 | 5 | 5.5 | 21.05 | 2.61 | 4.81 | 3.73 |
